# Impact of Ticagrelor vs. Clopidogrel in Patients With Acute Coronary Syndrome Undergoing Percutaneous Coronary Intervention After Risk Stratification With the CHA_2_DS_2_-VASc Score

**DOI:** 10.3389/fcvm.2022.808571

**Published:** 2022-04-04

**Authors:** Kun Na, Miaohan Qiu, Sicong Ma, Yi Li, Jing Li, Rong Liu, Jiaoyang Zhang, Yaling Han

**Affiliations:** ^1^School of Life Science and Biochemistry, Shenyang Pharmaceutical University, Shenyang, China; ^2^Department of Cardiology, General Hospital of Northern Theater Command, Shenyang, China; ^3^School of Graduate, Second Affiliated Hospital of Dalian Medical University, Dalian, China; ^4^School of Graduate, The Second Hospital of Jilin University, Changchun, China; ^5^School of Graduate, Second Affiliated Hospital of Harbin Medical University, Harbin, China

**Keywords:** P2Y_12_ receptor inhibitor, percutaneous coronary intervention, CHA_2_DS_2_-VASc score, acute coronary syndrome, prognosis

## Abstract

**Backgrounds:**

The clinical benefit of ticagrelor vs. clopidogrel in unselected patients with acute coronary syndrome (ACS) after percutaneous coronary intervention (PCI) remains controversial in the real world. This study was aimed to investigate the impact of ticagrelor vs. clopidogrel in subjects with ACS without atrial fibrillation or flutter (AF) after PCI based on risk stratification using the CHA_2_DS_2_-VASc score.

**Methods:**

In 2016–2019, patients who underwent PCI with at least one stent implanted in the General Hospital of Northern Theater Command were classified as low- or high-risk groups according to the CHA_2_DS_2_-VASc score. Incidences of 12-month ischemia [cardiac death, myocardial infarction (MI), or stroke], all-cause death, Bleeding Academic Research Consortium (BARC) 2,3,5 bleeding, BARC 3,5 bleeding, and net adverse clinical events (NACEs) (all-cause death, MI, stroke, or BARC 3, 5 bleeding) with aspirin plus different P2Y_12_ inhibitors (clopidogrel or ticagrelor) were appraised among different risk groups. Propensity score matching (PSM) and Cox multivariate analysis were used to balance the groups.

**Results:**

A total of consecutive 17,037 patients with ACS were enrolled. The optimal cut-off value of the CHA_2_DS_2_-VASc score for ischemic events by the Youden test was 3 points. Among patients with high risk (CHA_2_DS_2_-VASc ≥ 3, *n* = 6,151), ticagrelor was associated with slightly lower risks of ischemic events (2.29% vs. 3.54%, *P* = 0.02) and stroke (0.39% vs. 1.08%, *P* = 0.01) without excessive risk of BARC 3, 5 bleeding events (2.16% vs. 2.11%, P = 0.92) compared to clopidogrel within 12 months after PCI. For patients with low risk (CHA_2_DS_2_-VASc < 3, *n* = 10,886), a statistically significant difference was seen in the incidence of overall 12-month BARC 2, 3, 5 bleeding events by P2Y_12_ receptor inhibitor (4.00% vs. 3.26%) with a similar incidence of the ischemic events (1.40% vs. 1.52%). Results in the PSM cohort and the adjustment with Cox multivariate analysis were consistent with the main outcomes.

**Conclusion:**

Higher CHA_2_DS_2_-VASc scores were associated with a higher incidence of 1-year ischemic events for the patients with ACS after PCI. Compared with clopidogrel, ticagrelor was associated with lower ischemic events within 12 months after PCI without excessive risk of bleeding in high-risk patients but shows poor safety with excess bleeding in low-risk patients.

## Introduction

Dual antiplatelet therapy (DAPT), as the cornerstone of secondary prevention of recurrent ischemic events, is critically important for the long-term prognosis of patients with acute coronary syndrome (ACS) after percutaneous coronary intervention (PCI) (1, 2). With the emergence and widespread application of potent oral P2Y_12_ inhibitors, the combination of ticagrelor and aspirin, which could produce more powerful platelet inhibition and effectively reduce adverse cardiovascular events, is now broadly recommended by the current guidelines (3). However, the unavoidable concern about the increased bleeding risk associated with more potent antiplatelet strategies (4, 5) creates a dilemma regarding the balance of ischemic and bleeding events. Therefore, individualized antiplatelet therapy is urgently needed and remains unclear.

Risk-assessment tools built upon objective clinical characteristics are recommended by the current guidelines for accurately evaluating the prognosis of patients with ACS and guiding clinical practice (1). The Global Acute Coronary Event Registration (GRACE) score has shown the good capability of predicting the response of patients with ACS to various treatment modalities (6). However, due to the complexity of calculating the findings, which utilize numerous variables, including electrocardiography (ECG), cardiac biomarkers, etc., it is rather inconvenient to be used in daily operations. The CHA_2_DS_2_-VASc score, a refinement form of CHADS_2_ score, is widely applied to predict the risk of subsequent thromboembolic events in patients with atrial fibrillation (AF) (7). Surprisingly, a series of studies have demonstrated that a high CHA_2_DS_2_-VASc score was also remarkably associated with increased cardiocerebrovascular mortality in non-AF patients with ACS (8, 9). Additionally, CHA_2_DS_2_-VASc score, as a rapid and simple method of risk stratification, could be timely and conveniently obtained by physicians. Thus, we assumed that the CHA_2_DS_2_-VASc score, as a risk-assessment tool, may guide further optimal antithrombotic management such as the choice of P2Y_12_ inhibitors based on predicting prognosis in patients with ACS after PCI.

Therefore, we sought to investigate whether using the CHA_2_DS_2_-VASc score could improve the ability of discrimination to predict ischemic events in patients with ACS undergoing PCI and forwardly compare the efficacy and safety of ticagrelor vs. clopidogrel in a separate patient population stratified by CHA_2_DS_2_-VASc score in a real-world setting.

## Materials and Methods

### Data Sources and Population

This study was a *post-hoc* analysis of a single-center, all-comer, prospective, real-world PCI registry in the General Hospital of Northern Theater Command. Between March 2016 and March 2019, consecutive patients with ACS who underwent PCI were enrolled. In the present study, the inclusion criteria were patients with ACS ≥ 18 years of age, undergoing PCI with at least one stent implanted, and receiving clopidogrel- or ticagrelor-based DAPT. The exclusion criteria were as follows: (1) unavailable data for calculating CHA_2_DS_2_-VASc score; (2) switching between P2Y_12_ inhibitors during hospitalization; (3) diagnosis of AF during hospitalization. The study was approved by the hospital’s Research Ethics Committee with the agreement on an exemption from written informed consent (K2018-35). The study complied with the provisions of the Declaration of Helsinki. A standard web-based data collection platform (CV-NET system of Crealife Technology, Beijing, China) was used.

### Risk Assessment and Antiplatelet Strategy

The CHA_2_DS_2_-VASc score is a validated clinical prediction tool commonly used to estimate the risk of stroke in atrial fibrillation (AF), which was calculated by 2 points for a history of stroke or age ≥75 years, and 1 point for each of the following components: congestive heart failure (CHF), hypertension, type-2 diabetes mellitus, vascular disease, age 65–74 years, and female gender (7). All the patients were divided into two groups according to the optimal cutoff value of the CHA_2_DS_2_-VASc score.

The choice of antiplatelet strategy was left at the physician’s discretion. Before the procedure, patients were typically given a loading dose of aspirin (200–300 mg) and a P2Y_12_ inhibitor (either clopidogrel 300–600 mg or ticagrelor 180 mg). All the patients were discharged with a prescription for aspirin (100 mg daily) to be taken indefinitely, as well as clopidogrel (75 mg daily) or ticagrelor (90 mg twice daily) for at least 12 months. Optimal pharmacological therapy, including statins, angiotensin-converting enzyme inhibitor/angiotensin receptor blockers (ACEI/ARB), beta blockers, and proton pump inhibitors (PPI), was recommended at the discretion of the clinicians.

### Outcomes and Definitions

The primary endpoint was ischemic events at 12 months, defined as a composite of cardiac death, myocardial infarction (MI), or stroke. Secondary endpoints included each component of the primary endpoint, all-cause death, net adverse clinical events (NACEs), including all-cause death, MI, stroke, and Bleeding Academic Research Consortium (BARC) type 3, 5 bleeding events. The safety endpoint was BARC type 2, 3, 5 and type 3, 5 bleeding events. MI was defined according to the Third Universal Definition of Myocardial Infarction Guidelines (10). Stroke was defined as a loss of neurologic function induced by an ischemic episode that lasted at least 24 h or resulted in mortality, as determined by clinicians or imaging investigations. All-cause death was defined according to the Academic Research Consortium criteria (11). All patients were followed-up by telephone or email at 1, 6, and 12 months.

### Statistical Analysis

Continuous variables were presented as mean ± standard deviation (SD) and compared by Student’s *t*-tests. Categorical variables were presented as frequencies and percentages and compared using χ2 tests or Fisher exact tests. The Cochran–Armitage trend test was used for the trend of binary variables and the Mann–Kendall trend test for trends of continuous variables, and both were based on the CHA_2_DS_2_-VASc score. The Kaplan–Meier method was used to calculate time to clinical endpoints, and between-group differences were analyzed by the log-rank test. Hazard ratios and 95% confidence intervals (CI) were also calculated. The receiver-operating curve (ROC) was conducted to identify the discrimination of CHA_2_DS_2_-VASc score for assessing the ischemic risk among the cohort above, and the Youden test was used to obtain the optimal cut-off value that maximizes the sum of sensitivity and specificity, which was used to determine the cutoff value for ischemic risk stratification.

To minimize the bias of confounders on outcomes, propensity score matching analysis was performed in patients within CHA_2_DS_2_-VASc < 3 and CHA_2_DS_2_-VASc ≥ 3, separately. The propensity score used the nearest matching neighbor that included all the baseline clinical features, procedural characteristics, and medications, as shown in Table 1. The clinical features include age, sex, diabetes, hypertension, hyperlipidemia, smoking status, prior history of MI, stroke, PCI, coronary artery bypass graft (CABG) peripheral arterial disease (PAD), type of coronary artery disease (CAD), estimated glomerular filtration rate (eGFR), left ventricular ejection fraction (LVEF), anemia, and the medications are the use of aspirin, β-blockers, ACEI/ARB, statins, PPI. The procedural characteristics include target vessel location, *trans-*radial approach, stent diameter, number of stents per patient, total stent length per patient, and the SYNTAX score. Finally, the patients treated with ticagrelor were matched in a 1:1 ratio with patients treated with clopidogrel. As a sensitivity analysis, a Cox multivariate adjustment was made. Multivariate Cox analysis included factors with significant univariate differences (*P* < 0.05) or clinical relevance. A 2-sided *p*-value of <0.05 was considered to indicate statistical significance. Statistical analysis was performed using SAS software version 9.3 (SAS Institute, Cary, NC, United States).

**TABLE 1:** Clinical outcomes over 12 months according to the CHA_2_DS_2_-VASc score.

	CHA_2_DS_2_-VASc score					
	0 (*n* = 2,280)	1 (*n* = 4,441)	2 (*n* = 4,165)	3 (*n* = 2,947)	≥4 (*n* = 3,204)	*P*-value
Ischemic events	19(0.83%)	65(1.46%)	93(2.23%)	76(2.58%)	153(4.78%)	<0.01
Cardiac death	10(0.44%)	26(0.59%)	56(1.34%)	44(1.49%)	92(2.87%)	<0.01
MI	5(0.22%)	26(0.59%)	21(0.50%)	19(0.64%)	25(0.78%)	0.02
Stroke	5(0.22%)	15(0.34%)	23(0.55%)	15(0.51%)	40(1.25%)	<0.01
All-cause death	14(0.61%)	34(0.77%)	75(1.80%)	58(1.97%)	110(3.43%)	<0.01
BARC 2, 3, 5 bleeding events	74(3.25%)	161(3.63%)	150(3.60%)	97(3.29%)	121(3.78%)	0.58
BARC 3, 5 bleeding events	43(1.89%)	91(2.05%)	85(2.04%)	53(1.80%)	77(2.40%)	0.35

*Values are No. (%). p-values were calculated using the log-rank test based on all available follow-up data. Ischemic events, defined as a composite of cardiac death, myocardial infarction, or stroke. BARC indicates bleeding academic research consortium; MI, myocardial infarction.*

## Results

### Overview of the Study Population

**FIGURE 1:**
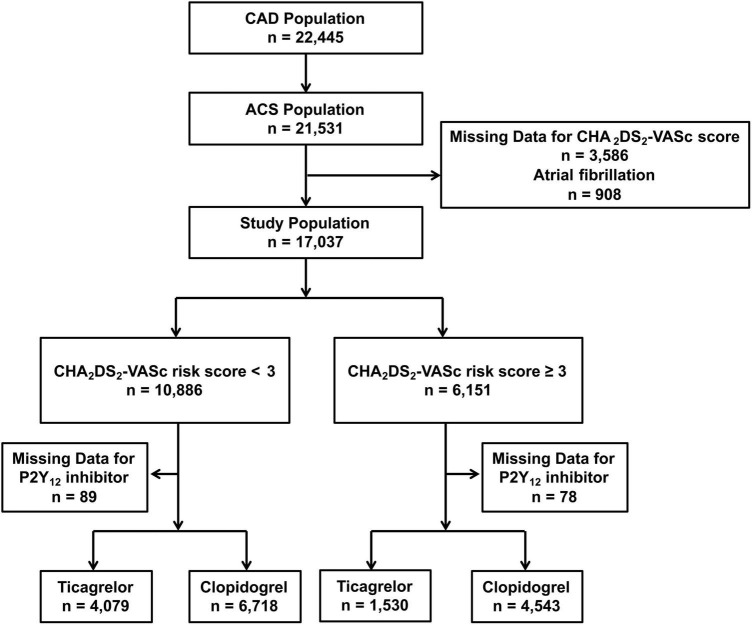
Flow chart of the study population.

A total of 17,037 patients with ACS without AF and undergoing PCI with a complete CHA_2_DS_2_-VASc score at discharge were divided into the low-risk group (CHA_2_DS_2_-VASc < 3, *n* = 10,886) and high-risk groups (CHA_2_DS_2_-VASc ≥ 3, *n* = 6,151). Among high-risk patients, 1,530/6,073 (25.2%) were treated with ticagrelor and the other 4,543/6,073 (74.8%) were treated with clopidogrel. Additionally, 4,079/10,797 (37.8%) low-risk patients were treated with ticagrelor and 6,718/10,797 (62.2%) were treated with clopidogrel. A flow chart for the study is shown in Figure 1. Patient characteristics, medication at discharge, and procedural features across various risk strata and according to different P2Y_12_ inhibitors are reported in the Supplementary Tables 1, 2, 4–7.

### Validation of the CHA_2_DS_2_-VASc Prediction Model

The distribution of patients with different CHA_2_DS_2_-VASc risk scores are shown in Supplementary Figure 1. The patients with the CHA_2_DS_2_-VASc score 1 reached the highest proportion, after which there was a gradual decline in distribution with an increase in the CHA_2_DS_2_-VASc risk score. The patients with CHA_2_DS_2_-VASc score>2 accounted for 36%. Higher scores were associated with higher incidence of 1-year ischemic events (0.83, 1.46, 2.23, 2.58, and 4.78% for patients with CHA_2_DS_2_-VASc scores of 0, 1, 2, 3, and ≥4, respectively, *p* < 0.01) (Table 1).

There was no statistically significant difference between the CHA_2_DS_2_-VASc score (area under the curve (AUC) 0.65, 95% CI: 0.62–0.68) and GRACE score (AUC 0.67, 95% CI: 0.64–0.70), *p* = 0.37 (assessed on the same population) (Supplementary Figure 2). A cut-off value of 3 by the Youden test with a sensitivity of 0.56 and a specificity of 0.64 was found in the CHA_2_DS_2_-VASc score to optimally categorize patients into groups at different ischemic risks.

Compared to the low-risk group (CHA_2_DS_2_-VASc < 3), the incidence of ischemic events (1.63% vs. 3.72%, *p* < 0.01) at 12 months was significantly higher in the high-risk group (CHA_2_DS_2_-VASc ≥ 3). Significant differences were also found in the incidence of the risk for MI (0.48% vs. 0.72%, *p* < 0.05), stroke (0.40% vs. 0.89%, *p* < 0.01), and cardiac (0.85% vs. 2.21%, *p* < 0.01) or all-cause death (1.13% vs. 2.73%, *p* < 0.01). There were no significant differences in the incidence of BARC 3, 5 bleeding (2.01% vs. 2.11%, *p* = 0.98) and BARC 2, 3, 5 bleeding (3.54% vs. 3.54%, *p* = 0.98) between the groups (Supplementary Figure 3 and Supplementary Table 3).

### Effects of Ticagrelor and Clopidogrel in the High-Risk Group

Among patients at high risk, patients with ticagrelor were younger (63.40 ± 8.11 vs. 68.37 ± 8.71, *p* < 0.01), with a higher rate of men (55.03% vs. 48.47%, *p* < 0.01) who were more likely to have previous MI (35.26% vs. 27.19%, *p* < 0.01) and PCI (37.32% vs. 32.61%, *p* < 0.01) as well as higher SYNTAX score (16.90 ± 9.56 vs. 15.97 ± 9.24, *p* < 0.01) than those treated with clopidogrel (Supplementary Tables 4, 5).

**FIGURE 2:**
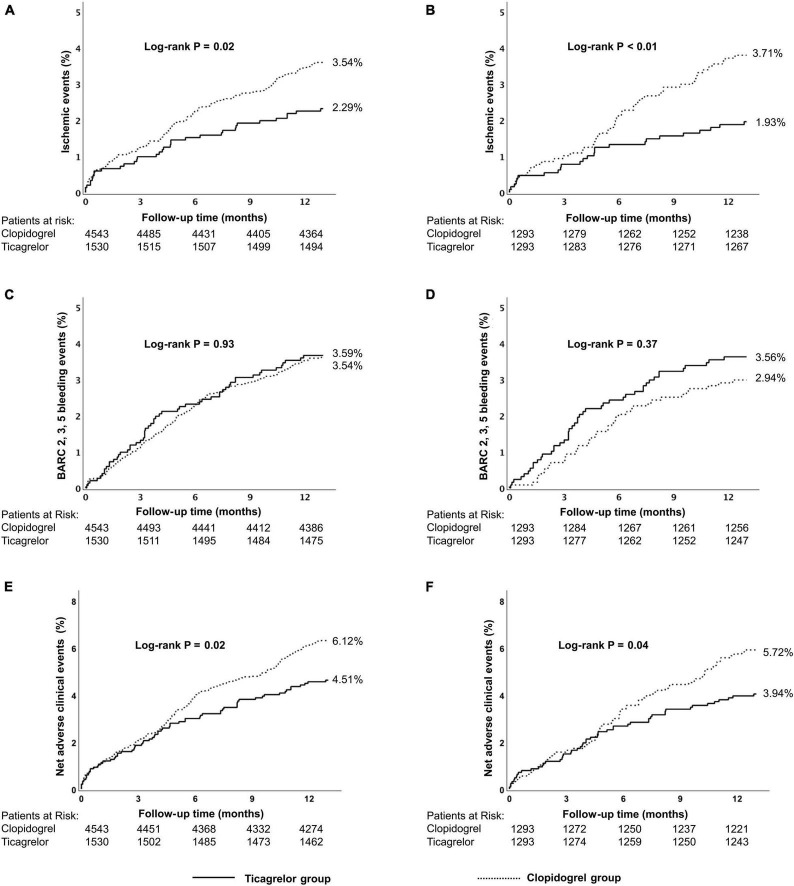
Kaplan–Meier cumulative event curves for a 12-month period for patients treated with ticagrelor or clopidogrel in the high-risk group, including primary endpoints ischemic events **(A,B)** BARC 2, 3, 5 bleeding **(C,D)** and net adverse clinical events (NACEs) **(E,F)**. Before propensity score matching **(A,C,E)**; after propensity score matching **(B,D,F)**. Ischemic events, defined as a composite of cardiac death, myocardial infarction (MI), and stroke.Net adverse clinical events, defined as a composite of all-cause death, myocardial infarction, stroke, and BARC type 3, 5 bleeding events. BARC, Bleeding Academic Research Consortium.

The incidence of the ischemic events was significantly lower in the ticagrelor group than that in the clopidogrel group (2.29% vs. 3.54%, *p* = 0.02) at 12 months. This significant difference was mainly derived from a significantly lower rate of stroke in the ticagrelor subgroup (0.39% vs. 1.08%, *p* = 0.01). The incidence of NACEs was significantly lower in the ticagrelor group than in the clopidogrel group (4.51% vs. 6.12%, *P* = 0.02). There were similar risks of BARC 2,3,5 bleeding (3.59% vs. 3.54%, *p* = 0.93) and BARC 3,5 bleeding (2.16% vs. 2.11%, *p* = 0.92) in the ticagrelor and clopidogrel group (Table 2 and Supplementary Figure 4). Kaplan–Meier curves are shown in Figure 2.

**TABLE 2:** Clinical outcomes over 12 months between clopidogrel and ticagrelor in the high-risk groups before and after propensity score matching.

	All patients			Propensity-matched patients		
	Ticagrelor (*N* = 1,530)	Clopidogrel (*N* = 4,543)	*P*-value	Ticagrelor (*N* = 1,293)	Clopidogrel (*N* = 1,293)	*P*-value
Ischemic events	35 (2.29%)	161 (3.54%)	0.02	25 (1.93%)	48 (3.71%)	<0.01
Cardiac death	21 (1.37%)	82 (1.80%)	0.26	14 (1.08%)	22 (1.70%)	0.18
MI	11 (0.72%)	33 (0.73%)	0.98	8 (0.62%)	6 (0.46%)	0.59
Stroke	6 (0.39%)	49 (1.08%)	0.01	6 (0.46%)	21 (1.62%)	<0.01
NACEs	69 (4.51%)	278 (6.12%)	0.02	51 (3.94%)	74 (5.72%)	0.04
All-cause death	24 (1.57%)	109 (2.40%)	0.05	17 (1.31%)	30 (2.32%)	0.06
BARC 2,3,5 bleeding events	55 (3.59%)	161 (3.54%)	0.93	46 (3.56%)	38 (2.94%)	0.37
BARC 3,5 bleeding events	33 (2.16%)	96 (2.11%)	0.92	26 (2.01%)	19 (1.47%)	0.29

*Values are No. (%). p-values were calculated using the log-rank test based on all available follow-up data. Ischemic events, defined as a composite of cardiac death, myocardial infarction, or stroke. Net adverse clinical events (NACEs), defined as a composite of all-cause death, myocardial infarction, stroke and BARC type 3, 5 bleeding events. BARC indicates bleeding academic research consortium; MI, myocardial infarction.*

Within the high-risk stratum, results in the propensity-matched cohort and the adjustment with Cox multivariate analysis were consistent with the main outcomes (Table 2 and Supplementary Table 8).

### Effects of Ticagrelor and Clopidogrel in the Low-Risk Group

For patients at low risk, compared with clopidogrel, ticagrelor was more frequently used in younger patients (55.35 ± 8.99 vs. 57.64 ± 8.86, *p* < 0.01), in men (89.53% vs. 84.62%, *p* < 0.01), and in patients with higher SYNTAX score (15.70 ± 8.96 vs. 13.97 ± 8.33, *p* < 0.01) (Supplementary Tables 6, 7).

**FIGURE 3:**
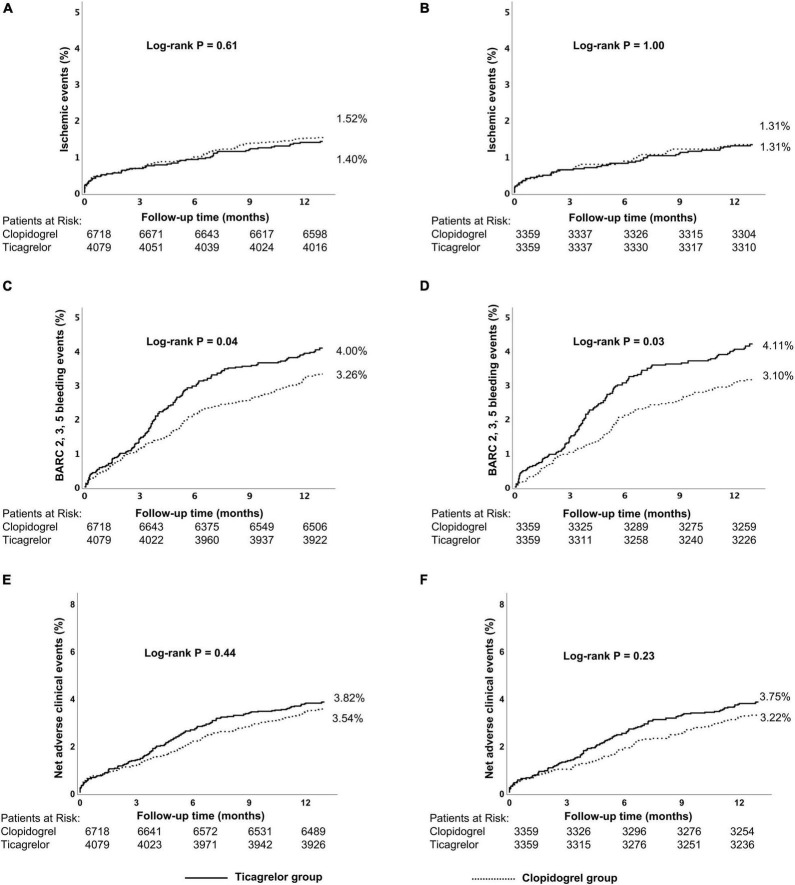
Kaplan–Meier cumulative event curves for a 12-month period for patients treated with ticagrelor or clopidogrel in the low-risk group, including primary endpoints ischemic events **(A,B)** BARC 2, 3, 5 bleeding **(C,D)** and net adverse clinical events (NACEs) **(E,F)**. Before propensity score matching **(A,C,E)**; after propensity score matching **(B,D,F)**. Ischemic events, defined as a composite of cardiac death, MI, and stroke.Net adverse clinical events (NACEs), defined as a composite of all-cause death, myocardial infarction, stroke, and BARC type 3, 5 bleeding events. BARC, Bleeding Academic Research Consortium.

There was no significant difference in the ischemic events (1.40% vs. 1.52%, *p* = 0.61) between ticagrelor and clopidogrel group as was for the risk for NACEs (3.82% vs. 3.54%, *p* = 0.44) or all-cause death (0.81 vs. 1.01%, *p* = 0.29), except for a significantly higher risk of BARC 2, 3, 5 bleeding events (4.00% vs. 3.26%, *p* = 0.04) (Table 2 and Supplementary Figure 4). Kaplan–Meier curves are shown in Figure 3 and Supplementary Figure 6.

Within the low-risk stratum, results in the propensity-matched cohort and the adjustment with Cox multivariable analysis were consistent with the main outcomes (Table 3 and Supplementary Table 9).

**TABLE 3:** Clinical outcomes over 12 months between clopidogrel and ticagrelor in the low-risk groups before and after propensity score matching.

	All patients			Propensity-matched patients		
	Ticagrelor (*N* = 4,079)	Clopidogrel (*N* = 6,718)	*P*-value	Ticagrelor (*N* = 3,359)	Clopidogrel (*N* = 3,359)	*P*-value
Ischemic events	57 (1.40%)	102 (1.52%)	0.61	44 (1.31%)	44 (1.31%)	1.00
Cardiac death	26 (0.64%)	48 (0.71%)	0.64	21 (0.63%)	22 (0.65%)	0.88
MI	21 (0.51%)	29 (0.43%)	0.54	15 (0.45%)	17 (0.51%)	0.72
Stroke	12 (0.29%)	31 (0.46%)	0.18	10 (0.30%)	8 (0.24%)	0.64
NACEs	156 (3.82%)	238 (3.54%)	0.44	126 (3.75%)	108 (3.22%)	0.23
All-cause death	33 (0.81%)	68 (1.01%)	0.29	27 (0.80%)	33 (0.98%)	0.44
BARC 2,3,5 bleeding events	163 (4.00%)	219 (3.26%)	0.04	138 (4.11%)	104 (3.10%)	0.03
BARC 3,5 bleeding events	93 (2.28%)	123 (1.83%)	0.11	77 (2.29%)	56 (1.67%)	0.07

*Values are No. (%). p-values were calculated using the log-rank test based on all available follow-up data. Ischemic events, defined as a composite of cardiac death, myocardial infarction, or stroke. Net adverse clinical events (NACEs), defined as a composite of all-cause death, myocardial infarction, stroke, and BARC type 3, 5 bleeding events. BARC indicates bleeding academic research consortium; MI, myocardial infarction.*

## Discussion

In this *post-hoc*, hypothesis-generating study, we first assessed the efficacy and safety of ticagrelor vs. clopidogrel stratified by the CHA_2_DS_2_-VASc score in patients with ACS undergoing PCI. The main results were as follows: (1) the CHA_2_DS_2_-VASc score demonstrated an acceptable clinical prediction value for risks of ischemic events and all-cause death at 12 months, and an optimal threshold of 3 points of CHA_2_DS_2_-VASc score was identified for the risk of ischemic events stratification of ACS patients with PCI. (2) In patients with CHA_2_DS_2_-VASc score ≥ 3, ticagrelor could reduce the risk of ischemic events compared with clopidogrel significantly without the expense of increased bleeding risk. This ischemic benefit of ticagrelor was not found among patients with CHA_2_DS_2_-VASc score < 3, which even increased the bleeding event rate.

Many observational studies have shown that the CHA_2_DS_2_-VASc score, using only a few demographic and clinical risk variables, was useful to estimate the risks of ischemic events or death in patients with ACS without AF (9, 12). A study that included 3,745 patients with ACS without AF who underwent PCI also found that a higher CHA_2_DS_2_-VASc score was associated with an increased incidence of major adverse cardiovascular events (MACEs) including cardiovascular death, non-fatal MI, or stroke, and the CHA_2_DS_2_-VASc score was an independent predictor of subsequent MACEs (HR: 1.31; 95% CI: 1.24–1.39, *p* < 0.001) (8). Another study enrolling 13,422 patients with ACS showed that a CHA_2_DS_2_-VASc score > 5 was associated with a fivefold increase in the risk of 1-year mortality compared to the low-risk group (CHA_2_DS_2_-VASc score 0–1), and even the intermediate group (CHA_2_DS_2_-VASc score 2–3) was also associated with increased 1-year mortality (13). Similarly, our study reported that among patients with ACS treated with PCI, the incidence of ischemic events and all-cause mortality also gradually increased with the elevation of the CHA_2_DS_2_-VASc score. All the components of CHA_2_DS_2_-VASc are important risk and prognostic factors for cardiovascular disease (14). These findings gave room for the application of the CHA_2_DS_2_-VASc score in clinical practice for physicians. The GRACE score is currently the only risk score with a class I recommendation in the non-ST-segment elevation myocardial infarction (NSTEMI) guidelines (1). Additionally, CHA_2_DS_2_-VASc score as an immediate, rapid, simple, and convenient method of risk stratification could guide clinical management even without using results of the following inspections, such as ECG parameters and cardiac biomarkers.

Establishing an optimal DAPT regimen is crucial for balancing ischemic and hemorrhage risks in ACS after PCI. The prevalence of cardiovascular risk factors in this Asian population is similar to that of other large contemporary trials (15) and real-world registries including other ethnicities (16). However, the concept of the “East Asian paradox”(17) is still critical in DAPT for the East Asian population, and the clinical benefit of ticagrelor in the unselected East Asian population is still doubtful. Similar to the results from the PHILO (4) (performed in Japan, South Korea, and Taiwan) and TICAKOREA (5) (performed in South Korea) trials, patients treated with ticagrelor compared to clopidogrel had a higher incidence of bleeding events and a higher, albeit statistically non-significant, incidence of the primary efficacy endpoint. Therefore, it is extremely important to identify specific patients who could practically benefit from ticagrelor. In our study, ROC analysis showed that patients with CHA_2_DS_2_-VASc score ≥ 3 were at higher risk of ischemic events. Furthermore, the THEMIS-PAD (18) trial also explored the efficacy and safety of ticagrelor vs. placebo among 19,220 patients with CAD and with comorbidities, such as diabetes and peripheral arterial disorder (PAD), whose CHA_2_DS_2_-VASc score corresponded to ≥2. At 3 years, ticagrelor gained long-term benefit for the THEMIS patients in preventing cardiovascular and limb events with the reduction of 20% for peripheral revascularization and about 50% for major adverse limb events, which was to some extent consistent with our results. According to a series of Platelet Inhibition and Patient Outcomes (PLATO) substudies, patients with ACS with age ≥ 75 years (19), diabetes (20), or prior stroke (21) may benefit more from ticagrelor than clopidogrel. The finding that ticagrelor reduced the risk of ischemic events without increasing the risk of bleeding in patients classified as high-risk may be explained by the CHA_2_DS_2_-VASc score’s incorporation of these risk variables. A consensus statement approves software like the VerifyNow system to help clinicians predict postoperative hemorrhage in antiplatelet therapy patients (22). Maybe it will further improve the value of the score. Therefore, as a simple risk stratification model, the CHA_2_DS_2_-VASc score might effectively identify a subgroup of patients with a high-risk profile of ischemic cardiovascular events who may benefit from ticagrelor treatment.

Current ACCF/AHA and ESC guidelines for DAPT recommend a ticagrelor regimen (90 mg twice daily) in addition to aspirin for patients with ACS after PCI (3). Despite these recommendations, several registries have shown that clopidogrel is more frequently used in real-world practice (23, 24). Additionally, based on the results of the Thrombin Receptor Antagonist for Clinical Event Reduction in Acute Coronary Syndrome (TRACER) experiment, increased attention has been paid to preventing bleeding complications in patients using DAPT. In the TRACER trial, which included 12,944 patients without STEMI, bleeding during follow-up (BARC type 2, 3, or 4) had a significantly higher mortality risk than patients who did not (25). Ticagrelor is a direct-acting, non-thienopyridine, reversible inhibitor of the P2Y12 receptor, which leads to consistent, more potent, and rapid inhibition of platelet function compared with clopidogrel (22, 26). However, more potent platelet inhibition increases the risk of bleeding. Two retrospective registry studies from South Korea reported that patients with ACS treated with ticagrelor had a significantly higher rate of bleeding events compared with those who received clopidogrel, without the reduction of major adverse cardiac and cerebrovascular event (MACCE), including cardiac death, MI, stent thrombosis, and stroke (5, 27). Likewise, we found that patients at low-risk using standard-dose ticagrelor experienced more bleeding events without a significant decrease in ischemic events and cardiac or all-cause mortality compared with clopidogrel.

Recently, it has been shown that the deescalation DAPT strategy, switching from ticagrelor 90 mg twice daily to 60 mg twice daily in patients with prior MI, was superior to the standard-dose ticagrelor continuing DAPT strategy in terms of TIMI major bleeding, although without statistical significance, whereas both doses of ticagrelor were found to significantly reduce major cardiovascular thrombotic events compared with a placebo (28), which made it possible for low-dose ticagrelor to be used as an alternative. When compared with clopidogrel, from a meta-analysis including 16 studies, low dose ticagrelor (60 mg, twice daily) in patients with ACS also significantly reduced the risk of MACEs defined by each study (OR: 0.39; 95% CI: 0.26–0.58, *p* < 0.01), and no significant difference of major bleeding events (OR: 1.16; 95% CI: 0.43–3.08, *p* = 0.77) was found between ticagrelor and clopidogrel (29). Therefore, low-dose ticagrelor may have the potential to be used as an alternative for long-term antiplatelet practice in patients with ACS without a relatively high ischemic risk. A growing emphasis is being placed on an individualized antiplatelet medication, such as adjusting the P2Y_12_ inhibitor. However, there is a shortage of effective standard-setting mechanisms. From our study, the CHA_2_DS_2_-VASc score showed a good value as a prediction model of ischemic risk but also could, to some degree, identify patients with ACS who may benefit from ticagrelor.

## Limitation

This study should be appraised in light of several limitations. First, the study was a *post-hoc* analysis based on a single-center Chinese PCI registry, thus carrying the limitations inherent to the kind of study design. Despite our results being adjusted by PSM and further verified by Cox analysis, other unknown and unmeasured confounders (i.e., DAPT adherence, concomitant drugs that have an influence on the clinical outcomes) could not be assessed. Second, we enrolled patients with ACS without known AF, but we cannot exempt them from the possibility of asymptomatic paroxysmal AF. However, several studies reported that the CHA_2_DS_2_-VASc score was independently associated with all-cause mortality in acute myocardial infarction patients irrespective of the presence of AF (28, 30). Third, the choice of antiplatelet strategy was left at the physician’s discretion rather than driven by CHA_2_DS_2_-VASc risk score prospectively, which might limit the clinical value. However, our study could offer a valuable reference for reflecting the current clinical practice and may further provide a basis for relevant research in the future.

## Conclusion

Higher CHA_2_DS_2_-VASc scores were associated with the higher incidence of 1-year ischemic events and stroke for the patients with ACS after PCI. Compared with clopidogrel, ticagrelor was associated with lower ischemic events within 12 months after PCI without excessive risk of bleeding in high-risk patients but shows poor safety with excess bleeding in low-risk patients. However, this deserves to be confirmed in adequately powered randomized studies.

## Data Availability Statement

The raw data supporting the conclusions of this article will be made available by the authors without undue reservation. Further inquiries can be directed to the corresponding author.

## Ethics Statement

The studies involving human participants were reviewed and approved by the ethics committee of Research Ethics Committee of General hospital of northern theater command. Written informed consent for participation was not required for this study in accordance with the national legislation and the institutional requirements.

## Author Contributions

KN and MQ designed the statistical plan, verified the underlying data, and was in charge of manuscript writing. YL designed the statistical plan and verified the underlying data. YL, KN, JL, SM, JZ, and RL contributed to recruiting subjects during the whole period of study. YH contributed to the leadership of the whole process of conducting the study and played the key role of initiating, designing, conducting, and concluding the study. All authors contributed to the article and approved the submitted version.

## Conflict of Interest

The authors declare that the research was conducted in the absence of any commercial or financial relationships that could be construed as a potential conflict of interest. The reviewer BZ declared a shared affiliation with two of the authors MQ and RL to the handling editor at time of review.

## Publisher’s Note

All claims expressed in this article are solely those of the authors and do not necessarily represent those of their affiliated organizations, or those of the publisher, the editors and the reviewers. Any product that may be evaluated in this article, or claim that may be made by its manufacturer, is not guaranteed or endorsed by the publisher.
