# Supplementary material for: Impact of Ticagrelor vs. Clopidogrel in Patients With Acute Coronary Syndrome Undergoing Percutaneous Coronary Intervention After Risk Stratification With the CHA2DS2-VASc Score
Source: Front Cardiovasc Med. 2022 Apr 4;9:808571. doi: 10.3389/fcvm.2022.808571 (PMC9013766; doi:10.3389/fcvm.2022.808571)
Supplement: Supplementary file 1 [file Data_Sheet_1.docx]

**SUPPLEMENTAL MATERIAL**

**CONTENT**

1. **Supplemental Methods: None**
2. **Supplemental Tables: 10**

Supplementary Table 1. Baseline clinical characteristics and medication after discharge in the low-risk and high-risk groups according to the CHA2DS2-VASc score

Supplementary Table 2. Comparison of lesion characteristics and procedural results between low-risk and high-risk groups according to the CHA2DS2-VASc score

Supplementary Table 3. Clinical outcomes for the low-risk and high-risk groups over 12 months according to the CHA2DS2-VASc score

Supplementary Table 4. Baseline characteristics after discharge in patients with CHA2DS2-VASc≥3 between clopidogrel and ticagrelor groups before and after propensity score matching

Supplementary Table 5. Comparison of lesion characteristics and procedural results between patients treated with ticagrelor and clopidogrel in the high-risk group before and after propensity score matching

Supplementary Table 6. Comparison of baseline clinical characteristics and medication after discharge between patients treated with ticagrelor and clopidogrel in the low-risk group before and after propensity score matching

Supplementary Table 7. Comparison of lesion characteristics and procedural results between patients treated with ticagrelor and clopidogrel in the low-risk group before and after propensity score matching

Supplementary Tables 8 Unadjusted and Cox-adjusted hazard ratios for clinical outcomes over 12 months among patients with high risk

Supplementary Table 9. Unadjusted and Cox-adjusted hazard ratios for clinical outcomes over 12 months among patients with low risk

1. **Supplemental Figures and Figure Legends: 4**

Supplementary Figure 1. The distribution of patients with different CHA2DS2-VASc risk scores

Supplementary Figure 2. Receiver operating characteristic curves of ischemic events for CHA2DS2-VASc risk score

Supplementary Figure 3. Kaplan–Meier cumulative event curves over 12 months for patients receiving P2Y12 receptor inhibitor. Including ischemic events(a), all-cause mortality (b), BARC 2-5 bleeding (c), and BARC 3-5 bleeding (d). BARC indicates bleeding academic research consortium ischemic events, defined as the composite of cardiac death, myocardial infarction (MI), or stroke

Supplementary Figure 4. Clinical outcomes over 12 months between clopidogrel and ticagrelor in different groups before and after propensity score matching. Before propensity score matching (a); after propensity score matching (b). Ischemic events, defined as a composite of cardiac death, myocardial infarction (MI), and stroke. BARC, Bleeding Academic Research Consortium.

1. **Supplemental References: None**

**Supplemental Table 1.** **Baseline clinical characteristics and medication after discharge in the low-risk and high-risk groups according to the CHA2DS2-VASc score.**

|  | CHA2DS2-VASc<3 ( N=10886) | CHA2DS2-VASc≥3  (N = 6151) | P value | |
| --- | --- | --- | --- | --- |
| Age (years) | 56.77±8.99 | 67.1±8.8 | <0.01 | |
| Male | 9414 (86.48%) | 3083 (50.12%) | <0.01 | |
| Diabetes | 2190 (20.12%) | 3188 (51.83%) | <0.01 | |
| Hypertension | 5231 (48.05%) | 5291 (86.02%) | <0.01 | |
| Smoking status |  |  | <0.01 | |
| None | 3553 (32.76%) | 3633 (59.17%) |  |  |
| Current smoker | 5676 (52.33%) | 1628 (26.51%) |  |  |
| Ex-smoker | 1618 (14.92%) | 879 (14.32%) |  |  |
| Previous MI | 1382 (12.72%) | 1791 (29.23%) | <0.01 | |
| Previous stroke | 231 (2.12%) | 2148 (34.92%) | <0.01 | |
| Previous PCI | 2381 (21.88%) | 2064 (33.59%) | <0.01 | |
| Peripheral arterial disease | 44 (0.40%) | 154 (2.50%) | <0.01 | |
| Previous CABG | 94 (0.86%) | 129 (2.10%) | <0.01 | |
| Type of CAD |  |  | <0.01 | |
| UA | 6344 (58.28%) | 3975 (64.62%) |  | |
| NSTEMI | 1687 (15.50%) | 1022 (16.62%) |  | |
| STEMI | 2855 (26.23%) | 1154 (18.76%) |  | |
| eGFR, mL/min/1.73 m^2^ | 97±23 | 86±26 | <0.01 | |
| LVEF (%) | 59±8 | 57±10 | <0.01 | |
| Anemia^a^ | 1058 (10.51%) | 1326 (23.07%) | <0.01 | |
| Medications at discharge | | | |  |
| Aspirin | 10687 (98.17%) | 5992 (97.42%) | <0.01 | |
| P2Y12 Receptor Inhibitor |  |  | <0.01 | |
| Clopidogrel | 4079 (37.78%) | 1530 (25.19%) |  | |
| Ticagrelor | 6718 (62.22%) | 4543 (74.81%) |  | |
| β-blockers | 7384 (67.83%) | 4404 (71.60%) | <0.01 | |
| ACEI/ARB | 6809 (62.55%) | 4345 (70.64%) | <0.01 | |
| Statins | 10109 (92.86%) | 5683 (92.39%) | 0.26 | |
| Proton pump inhibitors | 4065 (37.34%) | 2282 (37.10%) | 0.75 | |

Values are mean ± SD or No. (%). Abbreviation: MI, myocardial infarction; PCI, percutaneous coronary intervention; CABG, coronary artery bypass graft; CAD, coronary artery disease; UA, unstable angina; STEMI, ST-segment–elevation myocardial infarction; NSTEMI, non–ST-segment–elevation myocardial infarction; eGFR, estimated glomerular filtration rate; LVEF, left ventricular ejection fraction; ACEI/ARB, angiotensin converting enzyme inhibitor/angiotensin II receptor blocker

1. Anemia was defined as hemoglobin <13g/dL for men or <12g/dL for women.

**Supplemental Table 2.** **Comparison of lesion characteristics and procedural results between low-risk and high-risk groups according to the CHA2DS2-VASc score.**

|  | CHA2DS2-VASc<3 (N=10886) | CHA2DS2-VASc≥3 (N=6151) | P value |
| --- | --- | --- | --- |
| Radial artery | 10226 (93.94%) | 5497 (89.37%) | <0.01 |
| Target vessel location |  |  |  |
| LM | 546 (5.02%) | 367 (5.97%) | <0.01 |
| LAD | 5836 (53.61%) | 3200 (52.02%) | <0.05 |
| LCX | 2515 (23.10%) | 1526 (24.81%) | 0.01 |
| RCA | 3995 (36.70%) | 2451 (39.85%) | <0.01 |
| Average stent diameter | 3.09±0.79 | 2.97±0.60 | <0.01 |
| Stent per patient | 1.52±0.87 | 1.58±0.92 | <0.01 |
| Total length of stent | 40.77±26.72 | 42.40±27.81 | <0.01 |
| SYNTAX score | 14.68±8.65 | 16.26±9.36 | <0.01 |

Note: Values are n (%) or mean ± SD.

Abbreviations: SYNTAX, synergy between PCI with TAXUS and cardiac surgery. LM, [left](javascript:;) [main](javascript:;) [coronary](javascript:;) [artery](javascript:;); LAD, [left](javascript:;) [anterior](javascript:;) [descending](javascript:;) [branch](javascript:;); LCX, [left](javascript:;) [circumflex](javascript:;) [artery](javascript:;); RCA, [right](javascript:;) [coronary](javascript:;) [artery](javascript:;).

**Supplemental Table 3.** **Clinical outcomes for the low-risk and high-risk groups over 12 months according to the CHA2DS2-VASc score.**

|  | CHA2DS2-VASc<3 ( N=10886) | CHA2DS2-VASc≥3  (N = 6151) | P value |
| --- | --- | --- | --- |
| Ischemic events | 177 (1.63%) | 229 (3.72%) | <0.01 |
| Cardiac death | 92 (0.85%) | 136 (2.21%) | <0.01 |
| MI | 52 (0.48%) | 44 (0.72%) | <0.05 |
| Stroke | 43 (0.40%) | 55 (0.89%) | <0.01 |
| All-cause death | 123 (1.13%) | 168 (2.73%) | <0.01 |
| BARC 2, 3, 5 bleeding events | 385 (3.54%) | 218 (3.54%) | 0.98 |
| BARC 3, 5 bleeding events | 219 (2.01%) | 130 (2.11%) | 0.65 |

Note: Values are n (%) or mean ± SD.

P-values were calculated using the log-rank test based on all available follow-up data. Ischemic events, defined as a composite of cardiac death, myocardial infarction, or stroke.

Abbreviations: MI, myocardial infarction; BARC, Bleeding Academic Research Consortium.

**Supplemental Table 4.** **Baseline characteristics after discharge in patients with CHA2DS2-VASc≥3 between clopidogrel and ticagrelor groups before and after propensity score matching.**

|  | All patients | | | Propensity-matched patients | | |
| --- | --- | --- | --- | --- | --- | --- |
|  | Ticagrelor  (N=1530) | Clopidogrel  (N=4543) | P value | Ticagrelor (N=1293) | Clopidogrel (N=1293) | P value |
| Age (years) | 63.40±8.11 | 68.37±8.71 | <0.01 | 63.42±8.00 | 63.75±8.56 | 0.30 |
| Male | 842 (55.03%) | 2202 (48.47%) | <0.01 | 702 (54.29%) | 679 (52.51%) | 0.36 |
| Diabetes | 906 (59.22%) | 2242 (49.35%) | <0.01 | 759(58.70%) | 733(56.69%) | 0.30 |
| Hypertension | 1343 (87.78%) | 3880 (85.41%) | 0.02 | 1143 (88.40%) | 1144 (88.48%) | 0.95 |
| Smoking status |  |  | <0.01 |  |  | 0.94 |
| None | 831 (54.53%) | 2754 (60.69%) |  | 717 (55.45%) | 715 (55.30%) |  |
| Current smoker | 466 (30.58%) | 1142 (25.17%) |  | 387 (29.93%) | 383 (29.62%) |  |
| Ex-smoker | 227 (14.90%) | 642 (14.15%) |  | 189 (14.62%) | 195 (15.08%) |  |
| Previous MI | 538 (35.26%) | 1230 (27.19%) | <0.01 | 442 (34.18%) | 432 (33.41%) | 0.68 |
| Previous stroke | 488 (31.90%) | 1639 (36.08%) | <0.01 | 411 (31.79%) | 426 (32.95%) | 0.53 |
| Previous PCI | 571 (37.32%) | 1480 (32.61%) | <0.01 | 456 (35.27%) | 450 (34.80%) | 0.80 |
| Peripheral arterial disease | 28 (1.83%) | 125 (2.75%) | <0.05 | 27 (2.09%) | 22 (1.70%) | 0.47 |
| Previous CABG | 24 (1.57%) | 99 (2.18%) | 0.14 | 19 (1.47%) | 14 (1.08%) | 0.38 |
| Type of CAD |  |  | 0.96 |  |  | 0.89 |
| UA | 991 (64.77%) | 2952 (64.98%) |  | 864 (66.82%) | 875 (67.67%) |  |
| NSTEMI | 255 (16.67%) | 762 (16.77%) |  | 216 (16.71%) | 208 (16.09%) |  |
| STEMI | 284 (18.56%) | 829 (18.25%) |  | 213 (16.47%) | 210 (16.24%) |  |
| eGFR, mL/min/1.73 m2 | 90±25 | 85±26 | <0.01 | 90±25 | 89±26 | 0.28 |
| LVEF (%) | 57±10 | 58±10 | <0.01 | 57±10 | 58±10 | 0.20 |
| Anemia^a^ | 280 (19.57%) | 1013 (23.86%) | <0.01 | 243 (18.79%) | 276 (21.35%) | 0.10 |
| Medications at discharge |  |  |  |  |  |  |
| Aspirin | 1506 (98.43%) | 4455 (98.06%) | 0.35 | 1287 (99.54%) | 1291 (99.85%) | 0.16 |
| β-blockers | 1142 (74.64%) | 3241 (71.34%) | 0.01 | 962 (74.40%) | 948 (73.32%) | 0.53 |
| ACEI/ARB | 1122 (73.33%) | 3206 (70.57%) | 0.04 | 964 (74.56%) | 964 (74.56%) | 1.00 |
| Statins | 1413 (92.35%) | 4246 (93.46%) | 0.14 | 1210 (93.58%) | 1207 (93.35%) | 0.81 |
| Proton pump inhibitors | 509 (33.27%) | 1767 (38.90%) | <0.01 | 436 (33.72%) | 441 (34.11%) | 0.84 |

Legend as in Supplementary Table 1.

**Supplemental Table 5.** **Comparison of lesion characteristics and procedural results between patients treated with ticagrelor and clopidogrel in the high-risk group before and after propensity score matching.**

|  | All patients | | | Propensity-matched patients | | |
| --- | --- | --- | --- | --- | --- | --- |
|  | Ticagrelor  (N=1530) | Clopidogrel  (N=4543) | P value | Ticagrelor  (N=1530) | Clopidogrel  (N=4543) | P value |
| Radial artery | 1366 (89.28%) | 4072 (89.63%) | 0.70 | 1171 (90.56%) | 1162 (89.87%) | 0.55 |
| Target vessel location |  |  |  |  |  |  |
| LM | 124 (8.10%) | 235 (5.17%) | <0.01 | 103 (7.97%) | 112 (8.66%) | 0.52 |
| LAD | 837 (54.71%) | 2327 (51.22%) | 0.02 | 707 (54.68%) | 706 (54.60%) | 0.97 |
| LCX | 417 (27.25%) | 1098 (24.17%) | 0.02 | 340 (26.30%) | 359 (27.76%) | 0.40 |
| RCA | 558 (36.47%) | 1859 (40.92%) | <0.01 | 492 (38.05%) | 476 (36.81%) | 0.52 |
| Average stent diameter | 2.97±0.64 | 2.96±0.59 | 0.64 | 2.98±0.67 | 2.97±0.66 | 0.68 |
| Stents per patient | 1.70±0.95 | 1.56±0.90 | <0.01 | 1.82±0.88 | 1.83±0.87 | 0.79 |
| Total length of stent | 46.06±28.78 | 41.65±27.20 | <0.01 | 49.48±27.19 | 49.58±26.95 | 0.92 |
| SYNTAX score | 16.90±9.56 | 15.97±9.24 | <0.01 | 16.31±9.34 | 16.78±9.44 | 0.20 |

Legend as in Supplementary Table 2.

**Supplemental Table 6.** **Comparison of baseline clinical characteristics and medication after discharge between patients treated with ticagrelor and clopidogrel in the low-risk group before and after propensity score matching.**

|  | All patients | | | Propensity-matched patients | | |
| --- | --- | --- | --- | --- | --- | --- |
|  | Ticagrelor (N=4079) | Clopidogrel (N=6718) | P value | Ticagrelor (N=3359) | Clopidogrel (N=3359) | P value |
| Age (years) | 55.35±8.99 | 57.64±8.86 | <0.01 | 55.69±8.68 | 55.68±8.94 | 0.96 |
| Male | 3652 (89.53%) | 5685 (84.62%) | <0.01 | 2981 (88.75%) | 2971 (88.45%) | 0.70 |
| Diabetes | 880 (21.57%) | 1288 (19.17%) | <0.01 | 709 (21.11%) | 728 (21.67%) | 0.57 |
| Hypertension | 1962(48.10%) | 3230 (48.08%) | 0.98 | 1619(48.20%) | 1619(48.20%) | 1.00 |
| Smoking status |  |  | <0.01 |  |  | 0.95 |
| None | 1202 (29.58%) | 2325 (34.73%) |  | 1026 (30.54%) | 1037 (30.87%) |  |
| Current smoker | 2266 (55.76%) | 3356 (50.13%) |  | 1840 (54.78%) | 1827 (54.39%) |  |
| Ex-smoker | 596 (14.67%) | 1013 (15.13%) |  | 493 (14.68%) | 495 (14.74%) |  |
| Previous MI | 551 (13.51%) | 821 (12.25%) | 0.06 | 445 (13.25%) | 445 (13.25%) | 1.00 |
| Previous stroke | 56 (1.37%) | 172 (2.56%) | <0.01 | 40 (1.19%) | 43 (1.28%) | 0.74 |
| Previous PCI | 915 (22.44%) | 1457 (21.70%) | 0.37 | 712 (21.20%) | 739 (22.00%) | 0.42 |
| Peripheral arterial disease | 15 (0.37%) | 28 (0.42%) | 0.70 | 12 (0.36%) | 11 (0.33%) | 0.83 |
| Previous CABG | 31 (0.76%) | 61 (0.91%) | 0.42 | 21 (0.63%) | 23 (0.68%) | 0.76 |
| Type of CAD |  |  | <0.01 |  |  | 0.30 |
| UA | 2138 (52.41%) | 4165 (62.00%) |  | 1854 (55.19%) | 1895 (56.42%) |  |
| NSTEMI | 690 (16.92%) | 983 (14.63%) |  | 570 (16.97%) | 524 (15.60%) |  |
| STEMI | 1251 (30.67%) | 1570 (23.37%) |  | 935 (27.84%) | 940 (27.98%) |  |
| eGFR, mL/min/1.73 m2 | 98±22 | 97±24 | <0.01 | 98±22 | 97±23 | 0.09 |
| LVEF (%) | 58±8 | 60±8 | 0.04 | 59±8 | 59±8 | 0.59 |
| Anemia^a^ | 359 (9.48%) | 669 (10.79%) | 0.04 | 319 (9.50%) | 348 (10.36%) | 0.24 |
| Medications at discharge |  |  |  |  |  |  |
| Aspirin | 4016 (98.46%) | 6626 (98.63%) | 0.46 | 3345 (99.58%) | 3349 (99.70%) | 0.41 |
| β-blockers | 2840 (69.62%) | 4523 (67.33%) | 0.01 | 2371 (70.59%) | 2379 (70.82%) | 0.83 |
| ACEI/ARB | 2642 (64.77%) | 4154 (61.83%) | <0.01 | 2206 (65.67%) | 2200 (65.50%) | 0.88 |
| Statins | 3779 (92.65%) | 6301 (93.79%) | 0.02 | 3163 (94.16%) | 3154 (93.90%) | 0.64 |
| Proton pump inhibitors | 1509 (36.99%) | 2547 (37.91%) | 0.34 | 1283 (38.20%) | 1290 (38.40%) | 0.86 |

Legend as in Supplementary Table 1.

**Supplemental Table 7.** **Comparison of lesion characteristics and procedural results between patients treated with ticagrelor and clopidogrel in the low-risk group before and after propensity score matching.**

|  | All patients | | | Propensity-matched patients | | |
| --- | --- | --- | --- | --- | --- | --- |
|  | Ticagrelor (N=4079) | Clopidogrel (N=6718) | P value | Ticagrelor (N=4079) | Clopidogrel (N=6718) | P value |
| Radial artery | 3827 (93.82%) | 6327 (94.18%) | 0.45 | 3169 (94.3%) | 3161 (94.1%) | 0.68 |
| Target vessel location |  |  |  |  |  |  |
| LM | 302 (7.40%) | 239 (3.56%) | <0.01 | 229(6.82%) | 196(5.84%) | 0.10 |
| LAD | 2237 (54.84%) | 3566 (53.08%) | 0.08 | 1866(55.55%) | 1835(54.63%) | 0.45 |
| LCX | 922 (22.60%) | 1574 (23.43%) | 0.32 | 770(22.92%) | 797(23.73%) | 0.44 |
| RCA | 1468 (35.99%) | 2490 (37.06%) | 0.26 | 1191(35.46%) | 1198(35.67%) | 0.86 |
| Average stent diameter | 3.11±0.88 | 3.07±0.74 | 0.03 | 3.09±0.60 | 3.09±0.94 | 0.80 |
| Stents per patient | 1.63±0.93 | 1.47±0.82 | <0.01 | 1.69±0.84 | 1.68±0.81 | 0.50 |
| Total length of stent | 44.28±28.40 | 39.04±25.27 | <0.01 | 45.86±26.04 | 45.40±25.83 | 0.47 |
| SYNTAX score | 15.70±8.96 | 13.97±8.33 | <0.01 | 15.35±8.66 | 15.13±8.69 | 0.29 |

Legend as in Supplementary Table 2.**Supplemental Table 8.** **Unadjusted and Cox-adjusted hazard ratios for clinical outcomes over 12 months among patients with high risk.**

|  | Unadjusted HR (95% CI) | P for unadjusted HR | Adjusted HR (95% CI) | P for adjusted HR |
| --- | --- | --- | --- | --- |
| Ischemic events | 0.65 (0.46-0.94) | 0.02 | 0.65 (0.44-0.95) | 0.03 |
| NACEs | 0.73 (0.56-0.95) | 0.02 | 0.76 (0.57-1.00) | 0.046 |
| All-cause death | 0.67 (0.43-1.04) | 0.07 | 0.66 (0.42-1.05) | 0.08 |
| BARC 2, 3, 5 bleeding events | 1.01 (0.75-1.38) | 0.93 | 1.09 (0.79-1.50) | 0.60 |
| BARC 3, 5 bleeding events | 1.02 (0.69-1.51) | 0.92 | 1.12 (0.74-1.68) | 0.60 |

HR: Hazard ratio; 95%CI: 95% confidence intervals. Cox adjustment was performed for age, sex, smoking status, diabetes, hypertension, prior MI, prior stroke, prior PCI, prior CABG, type of CAD, eGFR, LVEF, anemia, β-blockers, ACEI/ARB, statins, target vessel location, average stent diameter, stents per patient, total length of stent, SYNTAX score. Ischemic events, defined as a composite of cardiac death, myocardial infarction, or stroke. Net adverse clinical events (NACEs), defined as a composite of all-cause death, myocardial infarction, stroke and BARC type 3, 5 bleeding events. BARC indicates bleeding academic research consortium; MI, myocardial infarction; eGFR, estimated glomerular filtration rate; LVEF, left ventricular ejection fraction; ACEI/ARB, angiotensin converting enzyme inhibitor/angiotensin II receptor blocker; SYNTAX, synergy between PCI with TAXUS and cardiac surgery

**Supplemental Table 9.** **Unadjusted and Cox-adjusted hazard ratios for clinical outcomes over 12 months among patients with low risk.**

|  | Unadjusted HR (95% CI) | P for unadjusted HR | Adjusted HR (95% CI) | P for adjusted HR |
| --- | --- | --- | --- | --- |
| Ischemic events | 0.92 (0.66-1.27) | 0.61 | 0.87 (0.62-1.21) | 0.41 |
| NACEs | 1.08 (0.88-1.32) | 0.44 | 1.08 (0.88-1.33) | 0.46 |
| All-cause death | 0.80 (0.53-1.21) | 0.29 | 0.75 (0.49-1.15) | 0.19 |
| BARC 2, 3, 5 bleeding events | 1.23 (1.01-1.51) | 0.04 | 1.26 (1.02-1.56) | 0.03 |
| BARC 3, 5 bleeding events | 1.25 (0.95-1.64) | 0.11 | 1.30 (0.98-1.71) | 0.07 |

Legend as in Supplementary Table 8.


Figure S1. The distribution of patients with different CHA2DS2-VASc risk scores


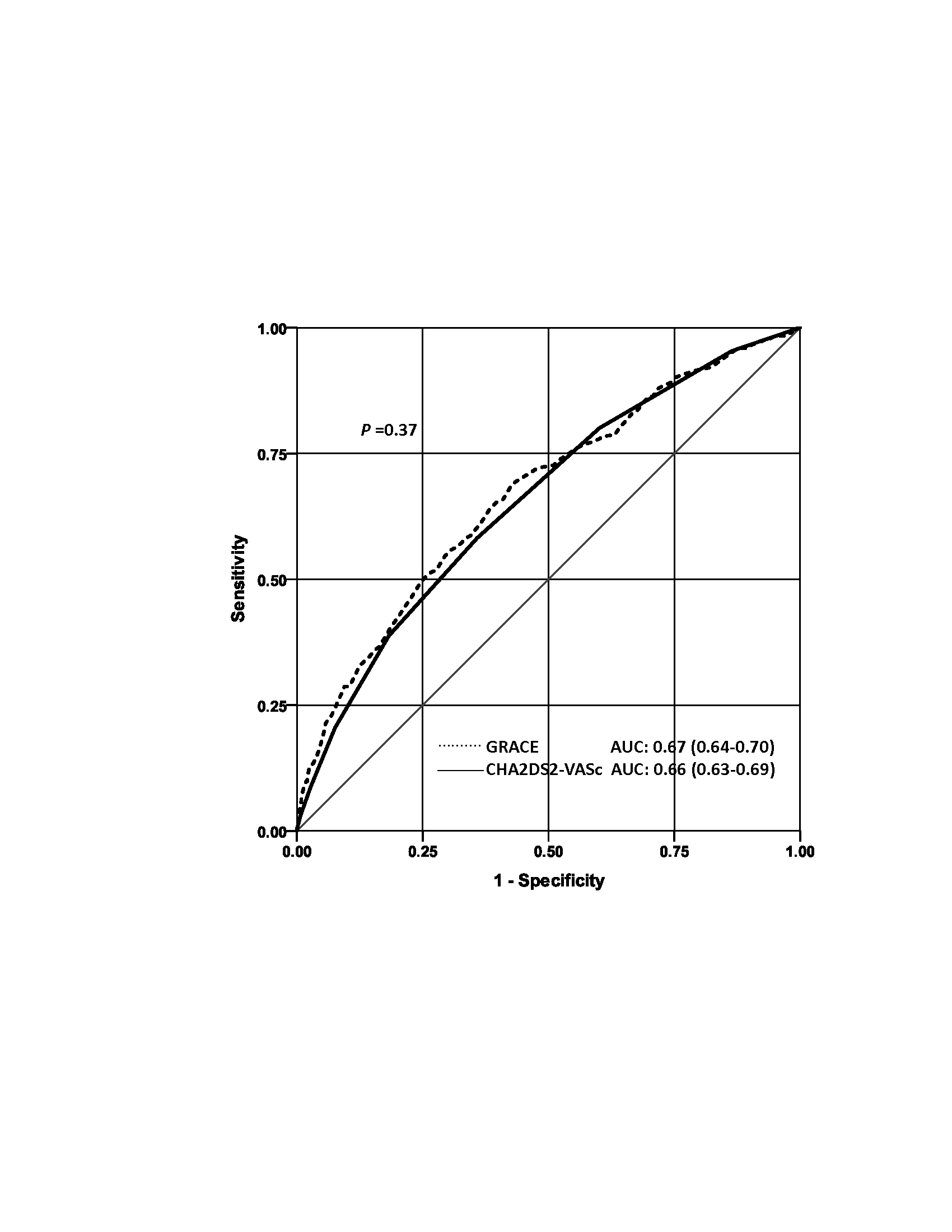


Figure S2. Receiver operating characteristic curves of ischemic events for CHA2DS2-VASc risk score


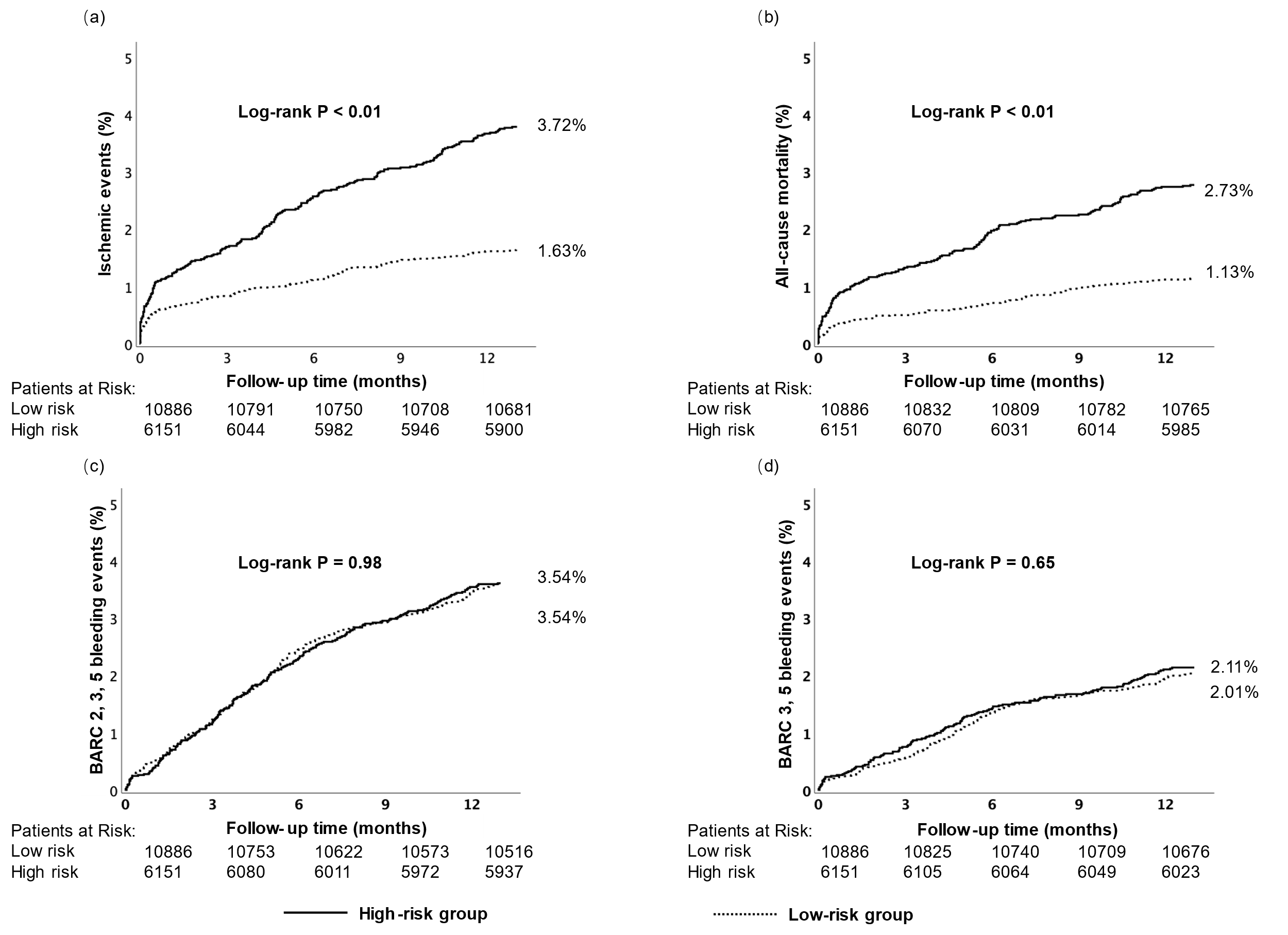


Figure S3. Kaplan–Meier cumulative event curves over 12 months for patients receiving P2Y12 receptor inhibitor. Including ischemic events(a), all-cause mortality (b), BARC 2-5 bleeding (c), and BARC 3-5 bleeding (d). BARC indicates bleeding academic research consortium ischemic events, defined as the composite of cardiac death, myocardial infarction (MI), or stroke.


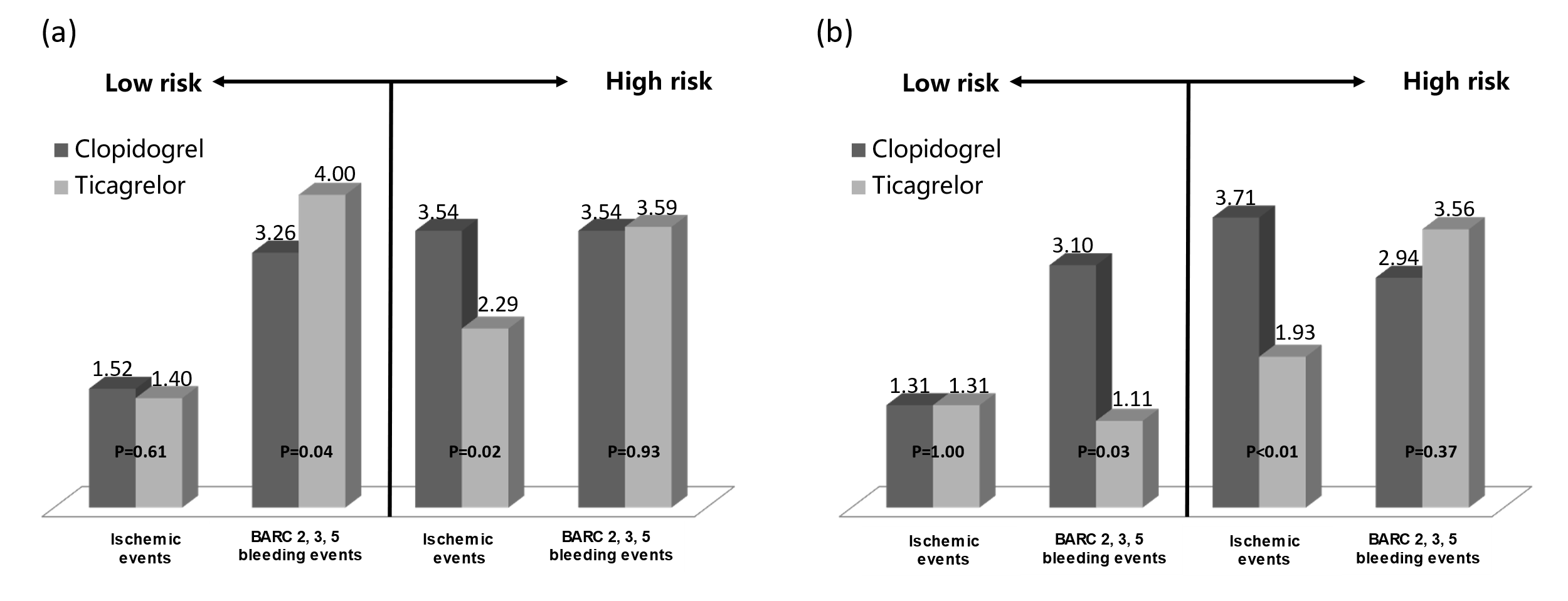


Figure S4. Clinical outcomes over 12 months between clopidogrel and ticagrelor in different groups before and after propensity score matching. Before propensity score matching (a); after propensity score matching (b). Ischemic events, defined as a composite of cardiac death, myocardial infarction (MI), and stroke. BARC, Bleeding Academic Research Consortium.
